# Supplementary material for: Validation of a Human Challenge Model Using an LT-Expressing Enterotoxigenic E. coli Strain (LSN03-016011) and Characterization of Potential Amelioration of Disease by an Investigational Oral Vaccine Candidate (VLA1701)
Source: Microorganisms. 2024 Apr 3;12(4):727. doi: 10.3390/microorganisms12040727 (PMC11051778; doi:10.3390/microorganisms12040727)
Supplement: Supplementary file 1 [file microorganisms-12-00727-s001.zip › microorganisms-2877589-supplementary.pdf]

Supplemental Tables and Figures for ETEC special issue manuscript

Inclusion exclusion criteria

Inclusion Criteria

Subjects who meet ALL of the following criteria are eligible for this study:

1. Healthy male and non-pregnant female subjects aged 18 to <50 years; health status is assessed by investigator at time of screening based on medical history, physical examination, and laboratory parameters.
2. BMI of 19.0 to 35.0 kg/m^2
3. Willingness to participate after informed consent has been obtained from the subject prior to any study related procedures.
4. Completion of a training session and demonstration of comprehension of the protocol procedures and knowledge of ETEC-associated illness by passing a written examination.
5. If subject is of childbearing potential:
    a) Negative pregnancy test at screening (Visit 0) with understanding to not become pregnant within 28 days after challenge;
    b) Subject has practiced an effective method of contraception (see below) during the 30 days before screening (Visit 0);
    c) Subject agrees to employ adequate birth control measures for the duration of the study. This includes one of the following measures:
        - Hormonal contraceptives (e.g. implants, birth control pills, patches);
        - Intrauterine device;
        - Barrier type of birth control measure (e.g. condoms, diaphragms, cervical caps);
        - Vasectomy in the male sex partner ≥3 months prior to first vaccination.

Exclusion Criteria

Subjects who meet ANY of the following criteria are NOT eligible for this study:

1. Participated in research involving investigational product within 30 days before planned date of first vaccination or planned use through Day 44;
2. Any prior exposure to ETEC (including LSN03-016011/A) or cholera occupationally or received LT (Or any mutant forms of LT (e.g., LTR192G, LTR192GL211A), ETEC, or cholera vaccine);
3. Subjects with known abnormal stooling patterns (fewer than 3 per week or more than 3 per day);
4. Known allergies to any component of the vaccine;
5. Subjects with known allergies to more than 1 planned antibiotics: Ciprofloxacin, Amoxicillin, trimethoprim-sulfamethoxazole;
6. History of diarrhea while traveling in a developing country within the last 3 years;
7. Subjects whose occupation involves handling of ETEC or cholera bacteria;
8. Women who are pregnant or breastfeeding;
9. Significant medical conditions including chronic, immunosuppressive, malignant, or gastrointestinal diseases (e.g. History of Irritable Bowel Syndrome (as defined by the Rome

III criteria or medical diagnosis) or gastric ulcer disease) or enteric, pulmonary, cardiac, liver or renal disease. Some medical conditions which are adequately treated and stable may be acceptable in the study (e.g. hypertension);

10. Significant abnormalities in screening lab hematology or serum chemistries, as determined by PI or PI in consultation with the independent Research Monitor and sponsor;
11. Use of any medication known to effect the immune system (e.g. systemic corticosteroids) within 30 days of vaccination or planned use during active study period (excluding inhaled steroids);
12. Evidence of confirmed infection with HIV, Hepatitis B or Hepatitis C;
13. Subjects with IgA deficiency (serum IgA < 7 mg/dl or limit of detection of assay);
14. Regular use of antacids, antidiarrheal, loperamide, bismuth subsalicylate, diphenoxylate or similar medication less than 2 weeks prior to enrolling in the study and through the inpatient portion of the study;
15. Known or suspected alcohol abuse or illicit drug use within the last year, positive urine toxicology for opioids, benzodiazepines or amphetamines;
16. Persons who are committed to an institution (by virtue of an order issued either by the judicial or the administrative authorities);
17. Persons who are in a dependent relationship with the sponsor, an investigator or other study team members, or the study center. Dependent relationships include close relatives and household members (i.e. children, partner/spouse, siblings, parents) as well as employees of the investigator or study center personnel;
18. Any other criteria which, in the investigator’s opinion, would compromise the ability of the subject to participate in the study, the safety of the study, or the results of the study.

Supplemental Figure S1: Study overview detailing the screening, enrollment, immunization, ETEC challenge and safety follow-up phases of the trial.

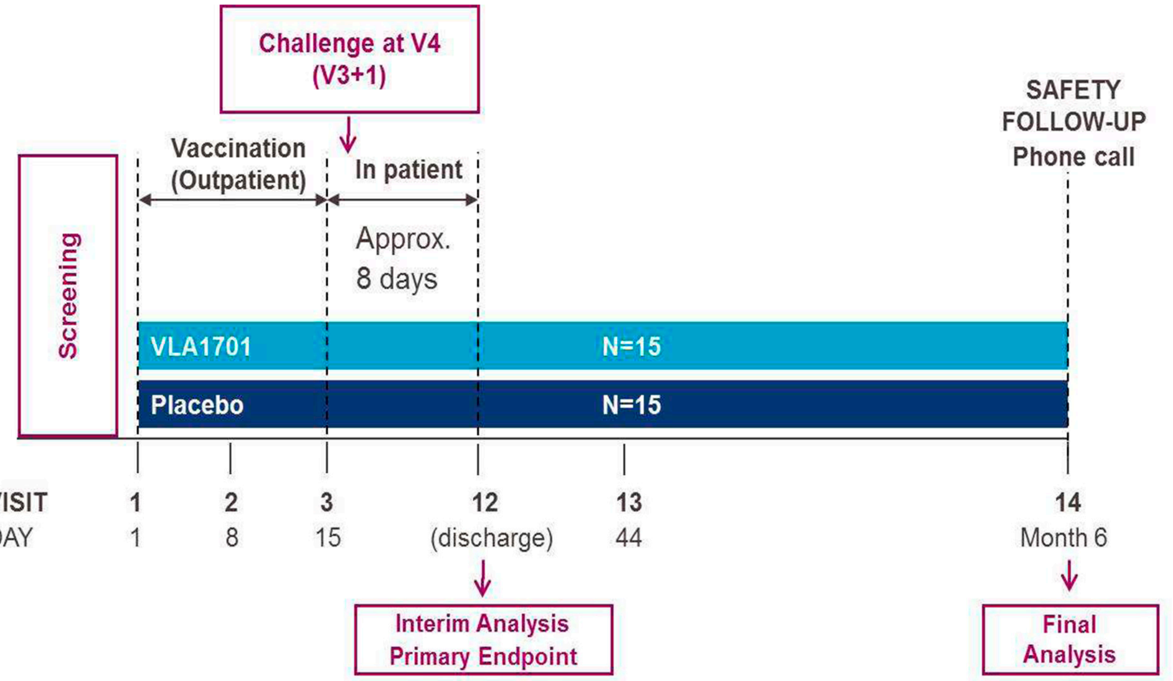

Supplemental Table S1: Outline of 3 component Disease Severity Score

Parameter	Outcome		Score
**Objective signs**	>1 episode of vomiting/24 hrs ***OR*** any fever		2
	1 episode of vomiting ***AND*** no fever		1
	No vomiting ***AND*** no fever		0
**Subjective symptoms**	Moderate-severe lightheadedness ***OR***		2
	Severe: nausea, malaise, headache or abd cramps		2
	Mild lightheadedness ***OR***		1
	mild-mod: nausea, malaise, headache or abd cramps		1
	No 'subjective symptoms'		0
**Diarrhea score (max 24 hr loose stools)**	>1000 ml	>12 episodes	4
	>600 to ≤1000 ml	>7 to 12 episodes	3
	>400 to ≤600 ml	>4 to ≤7 episodes	2
	>0 to ≤400 ml	1 to 4 episodes	1
	No loose stools	No loose stools	0

Footnote: diarrhea score assigned by the highest score determined by either maximum 24 hour output volume or frequency.

Supplemental Table S2. Demographics characteristics of subjects in the Phase 2B trial

Category		VLA1701	Placebo
	vaccinated	17	17
	challenged	15	15
Sex	male	14 (82.4%)	9 (52.9%)
	female	3 (17.6%)	8 (47.1%)
Race	Black/African American	16 (94.1%)	13 (76.5%)
	White	0 (0%)	2 (11.8%)
	American Indian/Black	1 (5.9%)	0 (0%)
	Other	0 (0%)	1 (5.9%)
Age	Mean	34.8	34.6
	Range	24-47	22-49

Supplemental Figure S2. Safety Assessment of the VLA 1701 ETEC/Cholera Vaccine

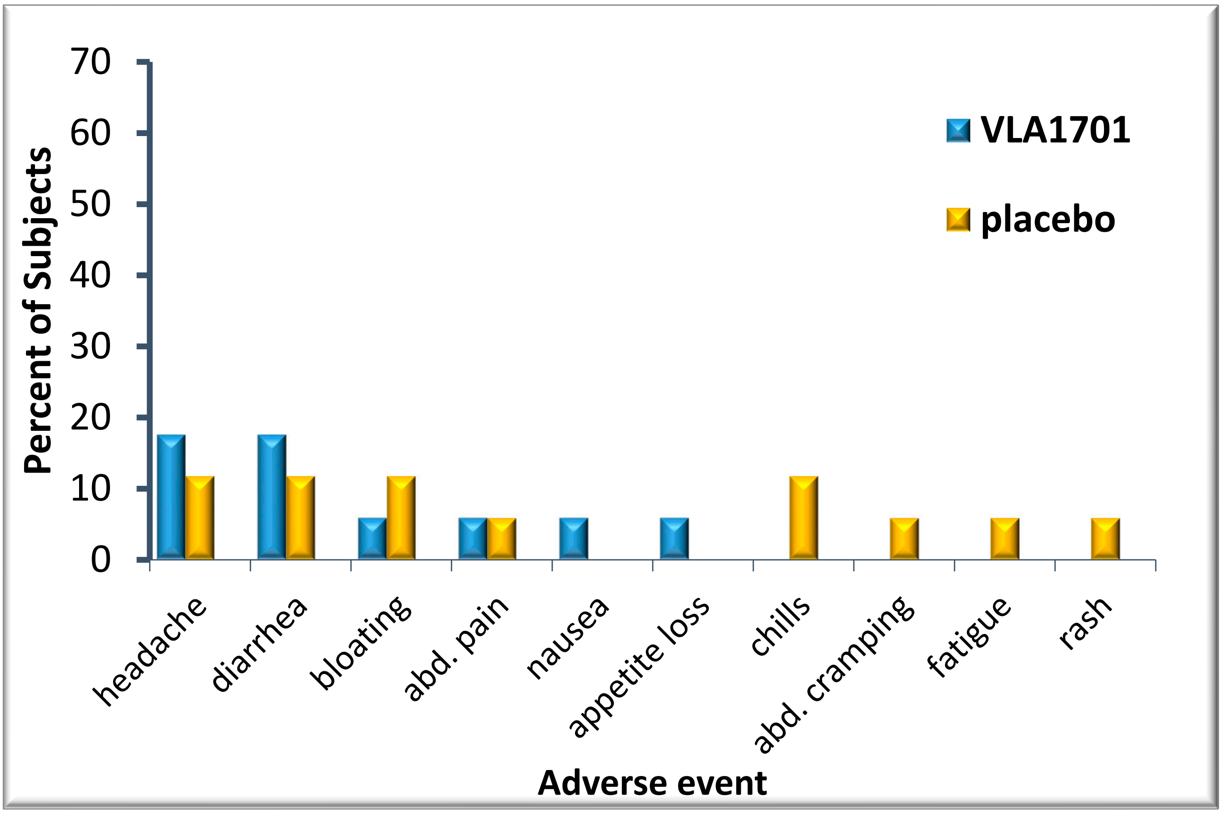

Note: All adverse events were mild, except 1 volunteer in the VLA1701 group that had moderate diarrhea after vaccination
